# Dengue Virus Type 3, Cuba, 2000–2002

**DOI:** 10.3201/eid1105.040916

**Published:** 2005-05

**Authors:** Rosmari Rodriguez-Roche, Mayling Alvarez, Edward C. Holmes, Lidice Bernardo, Gustavo Kouri, Ernest A. Gould, Scott Halstead, María G. Guzmán

**Affiliations:** * "Pedro Kouri" Tropical Medicine Institute, Havana, Cuba;; † University of Oxford, Oxford, UK;; ‡ Centre for Ecology and Hydrology, Oxford, UK;; § Pediatric Dengue Vaccine Initiative, Bethesda, Maryland, USA

**Keywords:** phylogenetic analysis, sequences, dengue viruses

**To the Editor:** In 1994, dengue virus type 3 (DENV-3) was reintroduced into Latin America after an absence of 17 years, and was isolated almost simultaneously in Nicaragua and Panama. In September 2000, DENV-3 was isolated for the first time in Cuba, producing a small outbreak that affected 3 areas of Havana City. In total, 138 cases of dengue fever were confirmed, with either DENV-3 or DENV-4 as the etiologic agents. No dengue hemorrhagic fever cases were observed, and the outbreak was brought under control within 6 weeks (*1*). At the end of June 2001, dengue was again reported in Havana. Through the end of February 2002, a total of 14,443 dengue cases were reported for the entire country, including 81 cases of dengue hemorrhagic fever and 3 fatalities (*2*).

To understand the molecular epidemiology of DENV-3 in Cuba, and particularly to determine whether the 2000 and 2001 outbreaks were caused by the same viral genotype, the complete envelope (E) gene sequences of isolates from both outbreaks were determined. To assist in this analysis, we also sequenced a DENV-3 strain representing the 1994 Nicaraguan epidemic, the first one isolated in Latin America in 1994 (*3*).

Three DENV-3 viruses isolated during the 2000–2002 Cuban outbreaks were studied. Two were obtained from acute-phase sera and the other from a spleen section sample from a patient who died. Serum samples and macerated spleen fragments were spread onto C6/36 mosquito cells (grown at 33°C) by using the rapid centrifugation assay (*4*).

Viral RNA was extracted from 200 μL of supernatant medium of virus-infected cells by using the RNAgents Total RNA Isolation system (Promega, Madison, WI, USA). The E gene was amplified by using reverse transcription–polymerase chain reaction (RT-PCR) as described previously (*5*). Double-stranded sequencing of the E gene was conducted on an ABI sequencer according to the manufacture's protocol (Applied Biosystems, Foster City, CA, USA). All sequences determined in this study have been deposited in GenBank (accession nos. AY702030–AY702033).

The E gene sequence of the 3 Cuban DENV-3 isolates and the single strain from Nicaragua isolated in 1994 were aligned with the E gene sequences (1,479 bp in length) of 60 DENV-3 isolates deposited in GenBank, representing the global genetic diversity of DENV-3. Phylogenetic trees were produced with a maximum likelihood method incorporating the GTR+ G+I model of nucleotide substitution, with the general time-reversible (GTR) substitution matrix, the base composition, the gamma (Γ) distribution of among-site rate variation, and the proportion of invariant sites (I) all estimated from the data. To explore the robustness of particular phylogenetic groupings, a bootstrap resampling analysis was undertaken. All analyses were performed with the PAUP* package (*6*).

The tree (Figure) showed 5 major groups or genotypes of DENV-3: 1) a Pacific/Asian group (genotype I); 2) an Asian group, containing a large array of viruses sampled from Thailand and Malaysia (genotype II); 3) a Latin American group that includes the Cuban viruses and isolates from Venezuela, Martinique, and Nicaragua, as well as those from Samoa, India, and Sri Lanka (genotype III); 4) a small group of Asian viruses (genotype IV); and 5) a final, most divergent group containing virus samples from Puerto Rico (genotype V). A similar genotype structure of DENV-3 has been observed in previous studies of this virus (*5**,**7**–**9*). The 3 Cuban viruses clearly fall into genotype III.

**Figure:**
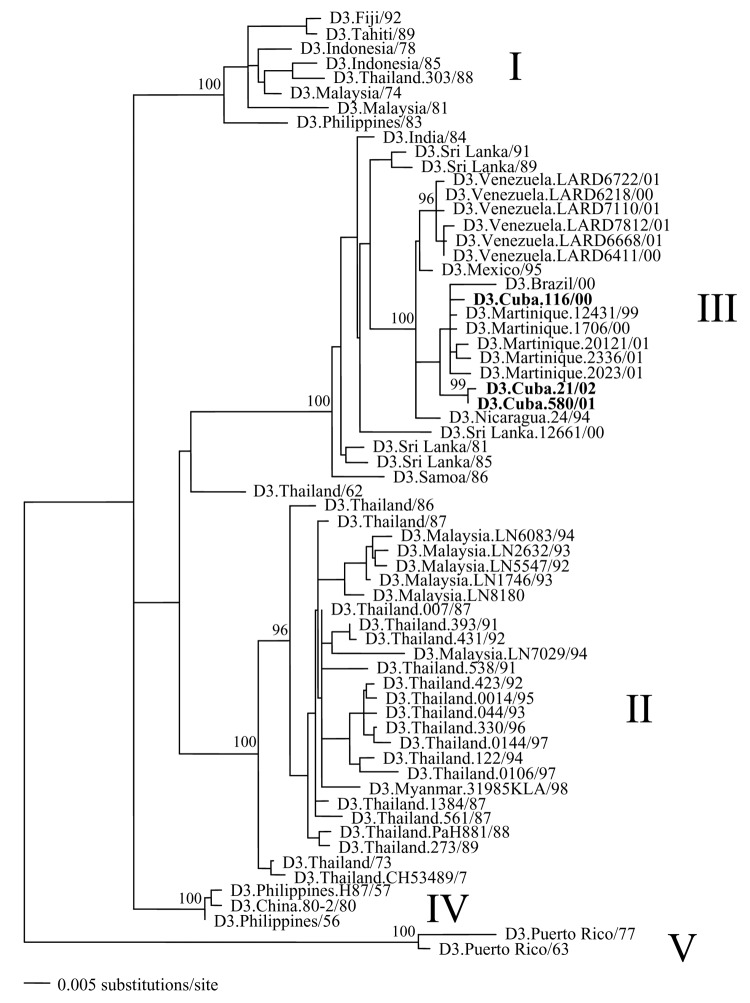
Maximum likelihood (ML) phylogenetic tree showing the evolutionary relationships among the E gene sequences of 64 strains of DENV-3. All branch lengths are drawn to scale, and tree was rooted using strains from the Puerto Rico 1960s epidemic, which always appear as the most divergent. Bootstrap support values are shown for key nodes only; the Cuban isolates are designated by bold type. See Table A1 for GenBank accession numbers of the other DENV-3 strains used in this analysis.

Indeed, strain D3.Nicaragua.24/94 falls at the base of the Latin American clade within genotype III, as would be expected if it was the first DENV-3 strain isolated from this region in 1994. Moreover, the most divergent strains from genotype III (with the exception of a single strain from Samoa) come from Asia, which suggests that this group of viruses was exported from Asia to Latin America in the early 1990s. Finally, the Cuban viruses fall into 2 distinct groups: the 116/00 strain, isolated during the 2000 outbreak, is very closely related to the Brazilian/Martinique viruses isolated in the same year, whereas strains 580/01 and 21/02, isolated in 2001–2002, appear to have diverged distinctly to form a separate cluster (99% bootstrap support).

A total of 15 nucleotide differences were distributed throughout the E gene. Of these, 12 were synonymous and 3 were nonsynonymous substitutions resulting in amino acid changes (E19 Thr/Pro, E226 Ile/Thr, and E329 Ala/Val). Moreover, 2 different nucleotide substitutions were found for the 2 isolates obtained during the major outbreak (2001–2002). These substitutions resulted in a nonconservative amino acid change (E22 Asp/Val). The ABI chromatograms of these sequences showed populations in this position, but the predominant population for each strain differed.

By using appropriate phylogenetic methods, deducing the most likely dispersal pattern for closely related dengue virus strains with different patterns of spatial and temporal sampling is possible. The current study strongly suggests that an Asian DENV-3 virus assigned to genotype III has evolved in situ in the Caribbean region since 1994.

The nucleotide differences observed in the Cuban isolates support the hypothesis that DENV-3 was introduced twice into Cuba from the wider Latin American region. Further evidence to support this hypothesis is that during the interepidemic period, from January to June 2001, no immunoglobulin (Ig) M–positive samples from patients with fever and suspected dengue cases were observed, which suggests that the virus was not circulating. Therefore, these data are consistent with the idea that, rather than in situ evolution, a second introduction of the virus occurred in 2001.

By comparing the amino acid sequences of the Cuban isolates with other DENV-3 strains assigned to genotype III, we confirmed that several distinct amino acid replacements had occurred. In particular, the nonconservative substitution Ala/Val was seen in the Nicaragua 1994, Mexico 1996, Cuba 2001–2002, and Venezuela 2001–2002 isolates. However, the Cuba 2000, Brazil 2000, and Martinique 1999–2001 isolates preserved the Ala at this position, as was also the case for viruses sampled from the putative Asian source (from India and Sri Lanka). The functional significance of amino acids at this position has not been determined.

## Appendix

**Table A1:** Dengue virus type 3 (DENV-3) strains used in phylogenetic analysis

DENV-3 strain	GenBank accession no.
D3 Brazil/00	AY038605
D3 China 80-2/1980	AF317645
D3 Fiji/92	L11422
D3 India/84	L11424
D3 Indonesia/78	L11426
D3 Indonesia/85	L11428
D3 Malaysia LN5547/92	AF147457
D3 Malaysia LN2632/93	AF147459
D3 Malaysia/74	L11429
D3 Malaysia/81	L11427
D3 Malaysia LN5547/92	AF147457
D3 Malaysia LN2632/93	AF147459
D3 Malaysia LN1746/93	AF147458
D3 Malaysia LN6083/94	AF147460
D3 Malaysia LN7029/94	AY338493
D3 Malaysia LN8180	AY338492
D3 Martinique 1243/99	AY099337
D3 Martinique 1706/00	AY099339
D3 Martinique 2012/01	AY099340
D3 Malaysia LN1746/93	AF147458
D3 Malaysia/81	L11427
D3 Malaysia LN6083/94	AF147460
D3 Malaysia/74	L11429
D3 Malaysia LN7029/94	AY338493
D3 Malaysia LN8180	AY338492
D3 Martinique 1243/99	AY099337
D3 Martinique 1706/00	AY099339
D3 Martinique 2012/01	AY099340
D3 Martinique 2023/01	AY099341
D3 Martinique 2336/01	AY099342
D3 Mexico 6097/95	AY146763
D3 Myanmar 31985KLA/98	AY145712
D3 Philippines/56	L11423
D3 Philippines H87/57	M93130
D3 Philippines/83	L11432
D3 Puerto Rico/63	L11433
D3 Puerto Rico/77	AY146761
D3 Samoa/86	L11435
D3 Sri Lanka/81	L11431
D3 Sri Lanka/85	L11436
D3 Sri Lanka/89	L11437
